# Effect of the menstrual cycle on common cold susceptibility and severity: insights from the Pittsburgh cold study 3 dataset

**DOI:** 10.1016/j.bbih.2026.101402

**Published:** 2026-11-04

**Authors:** Helen G. Hanstock, Sophie E. Harrison

**Affiliations:** a Swedish Winter Sports Research Centre, Department of Health Sciences, Mid Sweden University, Sweden; b Institute for Applied Human Physiology, School of Sport Science, Bangor University, Wales, UK

**Keywords:** Immune function, Rhinovirus, URTI, Premenopausal women, Menstrual cycle phase

## Abstract

Changes in circulating immune parameters over the menstrual cycle have been previously described, but there is limited evidence linking these changes to alterations in common cold susceptibility. Using data from the Pittsburgh Cold Study 3 we investigated whether menstrual cycle phase affects susceptibility to and severity of experimental rhinovirus infection. This secondary analysis included 27 naturally menstruating (NM) women and 17 hormonal contraception (HC) users <40 years. Cycle day and phase were estimated from self-reported data, whereby the first five days of the cycle were labelled as the early follicular (EF) phase, the final 10 days as the luteal (L) phase and remaining days as the transition period (TP). Dominant phase during the 5-day post-inoculation quarantine was taken as the mode for EF (n = 9) and L phases (n = 5) only. Objective cold development did not differ between NM (44%) and HC (35%; p = 0.549), nor between phase on quarantine day 1 within NM (χ^2^ = 0.89, p = 0.64). However, rhinovirus severity was lower during in L versus EF, represented by peak viral nasal titre (p = 0.003, d = 1.71), total symptom scores (p = 0.01, d = 1.37) and total adjusted mucus weight (p = 0.03, d = 0.86). These observations were supported by linear mixed models for viral load and symptoms during quarantine, which showed blunted viral load and symptom trajectories in L vs. EF. These exploratory, hypothesis generating findings suggest that menstrual cycle phase may influence common cold severity in healthy premenopausal women.

Viral respiratory tract infections, such as common colds, are the most common illnesses in humans and are accountable for considerable economic burden, accounting for 33 million days lost from work in the UK in 2024 (Fendrick et al., 2003; Office for National Statistics, 2025). Viral challenge studies have identified traits and lifestyle factors associated with illness risk, including smoking, low social integration, introversion, chronic stress, self-reported health, childhood socioeconomic status, and genetics (Cohen et al., 1997, 1998, 2013, 2015). Additionally, higher psychological stress, lack of sleep and lower physical activity levels have been associated with susceptibility to the common cold in both experimental and epidemiological research (Cohen et al., 1991; Ghilotti et al., 2018; Prather et al., 2015). At the systems level, the immune system appears to be shaped substantially by non-heritable influences as we age (Brodin et al., 2015), as well as showing seasonal changes (Dopico et al., 2015), further supporting the view that immunity and infection susceptibility may fluctuate within individuals over time.

The menstrual cycle, characterised by cyclical fluctuations in ovarian hormones, has been shown to modulate various aspects of immune function (Kadel and Kovats, 2018). The luteal phase, associated with higher oestradiol (E2) and progesterone concentrations, is associated with increased circulating innate immune cell counts (Notbohm et al., 2023). The possible clinical relevance of these interactions between ovarian hormones and immunity is illustrated by data from Covid-19, whereby higher E2 has been purported to be protective against infection (Ding et al., 2021). On the other hand, infection reactivation has been reported during menstruation, when ovarian hormone levels are low, and 30% of naturally menstruating long-Covid patients report symptom relapse directly before or during menstruation (Davis et al., 2021; Zheng et al., 2021). If ovarian hormones can modulate host defence against respiratory viruses, we would expect women to experience fluctuations in infection susceptibility or severity that recur cyclically as hormone levels rise and fall.

Experimental evidence of infection risk factors using controlled studies remains limited, with the majority of free-living studies not able to control exposure or confirm specific viral aetiologies of respiratory illnesses. The Common Cold Project (CCP; www.commoncoldproject.com), collated in 2011 from 25 years of historical studies utilising experimental rhinovirus inoculation in quarantine, provides a unique opportunity to examine factors associated with common cold susceptibility in a prospective, controlled manner. To this end, using CCP data, we investigated whether the menstrual cycle influences viral infection dynamics and clinical outcomes following experimental rhinovirus challenge.

## 1 Methods

The data were collected by the Laboratory for the Study of Stress, Immunity, and Disease at Carnegie Mellon University under the directorship of Sheldon Cohen, PhD; and were accessed via the Common Cold Project website (www.commoncoldproject.com; grant number NCCIH AT006694 (Laboratory for the Study of StressImmunityand Disease, 2016). We screened the CCP “Five Study” dataset for female participants, naturally menstruating or hormonal contraception users, with exclusion criteria >40 years of age or no longer having periods. Participants were considered naturally menstruating if they self-reported a regular menstrual cycle with an average length of between 21 and 35 days (Elliott-Sale et al., 2021); and no use of hormonal medication. Hormonal contraception users self-reported not using any other hormonal medications. All returned participants were contained in the PCS3 study dataset, tested between 2007 and 2011.

### 1.1 Participants

Female participants (n = 44) aged 24 ± 5 years were included: 27 naturally menstruating women (NM group) and 17 hormonal contraceptive users (HC group) (Fig. S1). In the HC group, 15 participants self-reported oral contraceptive pill (OCP) use, and two self-reported using a Levonorgestrel-releasing implant (LRI). No brand name or hormone concentration information was reported. Participants had returned negative pregnancy tests repeatedly between 7 and 8 weeks pre-quarantine until day 0 of quarantine.

### 1.2 Study methodology

For full methodology of the study design and collection of the outcome measures, readers are referred to the original PCS3 study (Doyle et al., 2010) and the CCP website. Briefly, participants were admitted to quarantine on day 0 and underwent a battery of tests, including reporting baseline symptoms and providing nasal lavage samples. At ∼17:00 on quarantine day 0, participants were administered nasal drops containing a standardised dose of rhinovirus 39. Participants were then monitored in quarantine for five days for development of infection, and objective signs of illness. Daily nasal lavage samples were obtained and used for viral quantification (day 1-5) (Gwaltney et al., 1989). Participants reported common cold symptoms daily during quarantine using the Jackson Common Cold questionnaire (Jackson et al., 1958).

Appropriate outcome measures and predictor variables related to common cold susceptibility, severity and health status were selected using screening of the study codebook and previous publications from the project. Common cold susceptibility was assessed by objective cold incidence. Participants were considered to have an objective common cold if the virus was recovered in nasal secretions at any time after inoculation, or if they displayed a 4-fold or greater increase in virus-specific antibodies at 28-days post-challenge, accompanied by clinical signs of upper respiratory tract involvement, defined as either a total baseline-adjusted mucus weight of 10g or more, or average baseline-adjusted mucociliary clearance time of ≥7 min (Cohen et al., 1997). Viral replication was assessed by daily nasal titres during quarantine, with peak nasal titres across the quarantine period used as a summary per-participant variable. Common cold severity was assessed both subjectively and objectively. Subjective common cold severity was assessed by daily Jackson symptom score and summarised as total Jackson symptom score during quarantine. Objective common cold severity was summarised as total mucous weight and mean mucociliary clearance time across the quarantine period.

### 1.3 Estimation of menstrual phases

Menstrual cycle phases were estimated by the research team using a calendar-based counting method, based on self-reported menstrual period dates collected before and after quarantine. The early follicular phase (EF) was estimated as the first five days of the cycle following onset of menstruation (Elliott-Sale et al., 2021; Ferrell et al., 2012); the luteal phase (L) as the final 10 days of the cycle before the next reported menstrual period (Bull et al., 2019). Neither ovarian hormone concentrations or ovulation were assessed; as such, phase labels should be considered estimates, and conditions such as luteal phase deficiency cannot be excluded. All interim days were labelled as a transitional period (TP), covering the transition from late follicular through peri-ovulatory, and possibly early luteal days, considered to represent a heterogenous ovarian hormone profile. This label represents a pragmatic approach to classify the remaining observations without anchoring to a specific hormonal milieu. Phase was determined for each given day of quarantine, with the dominant phase defined as the most common phase for each participant during quarantine.

### 1.4 Statistical analysis

To determine whether group (NM or HC) affected objective cold susceptibility, we used logistic regression to examine the association between menstrual cycle phase on quarantine day 1 (corresponding to the peak viral replication timing) and objective cold development. Effects of group on global objective and subjective common cold severity variables were assessed using Wilcoxon signed rank tests as well as Fisher's exact test for the proportion of participants who ever shed the virus. To compare objective cold susceptibility between phases (EF, TP and L) on day 1 within the NM group, we employed Firth penalised logistic regression using the *logistf* package, which provides more stable confidence intervals when counts are low, as was the case for the L phase. Overall significance of menstrual cycle group and phase was evaluated using a likelihood ratio test. Odds ratios (OR) and 95% confidence intervals (95% CI) are also reported for paired contrasts.

To explore effects of estimated phase (EF, TP, and L) on viral nasal titres and symptom scores, we constructed linear mixed-effects models for longitudinal data during quarantine. After checking model assumptions, viral nasal titre data were log-transformed again before analysis. Models were constructed with phase as a predictor on any given day of quarantine, including linear and quadratic day terms included to model trajectories. In the symptom score model, baseline symptom scores on day 0 (pre-inoculation) were included as a covariate. AIC/BIC criteria and R^2^ conditional and marginal results were used to inform model selection, with lower AIC and higher R^2^ marginal prioritised; in both cases, the quadratic models were favoured (details in Fig. S3). Type II omnibus tests were used to evaluate overall model terms. Fixed-effect coefficients (β estimates and 95% confidence intervals) and post-hoc contrasts are reported, with Benjamini-Hochberg correction applied separately within each model to account for multiple testing.

For follow-up analyses that aimed to specifically contrast participants tested predominantly in the EF vs L phases, a stricter classification was applied, whereby participants who transitioned between from L to EF during quarantine were removed (n = 2). Given the resultant low sample sizes, we conducted both frequentist and Bayesian t-tests with directional hypotheses (EF > L) and weak informative priors (Cauchy r = 0.5) to quantify evidence strength for the difference between phases. We additionally performed a leave-one-out sensitivity analysis for significant findings to determine the risk of results being affected by single influential points. BF_10_ values were interpreted using the classification scheme from Lee and Wagenmakers, as described by Quintana and Williams (2018). All analyses were conducted in R studio version 2024.12.1 with significance set at α = 0.05. Data are presented as mean ± SD, unless otherwise stated.

## 2 Results

Baseline characteristics were well-balanced between NM and HC, and estimated phases within NM (Table S1). Menstrual characteristics were similar between groups: menstruation and withdrawal bleed duration were 5 ± 1 days for each group. Within NM, self-reported cycle length was 28 ± 2 days and cycle length during the experimental period was 29 ± 4 days. Most participants reported good to excellent general health (84% overall). Phase distribution across quarantine days among NM participants is shown in Fig. 1A.

**Fig. 1:**
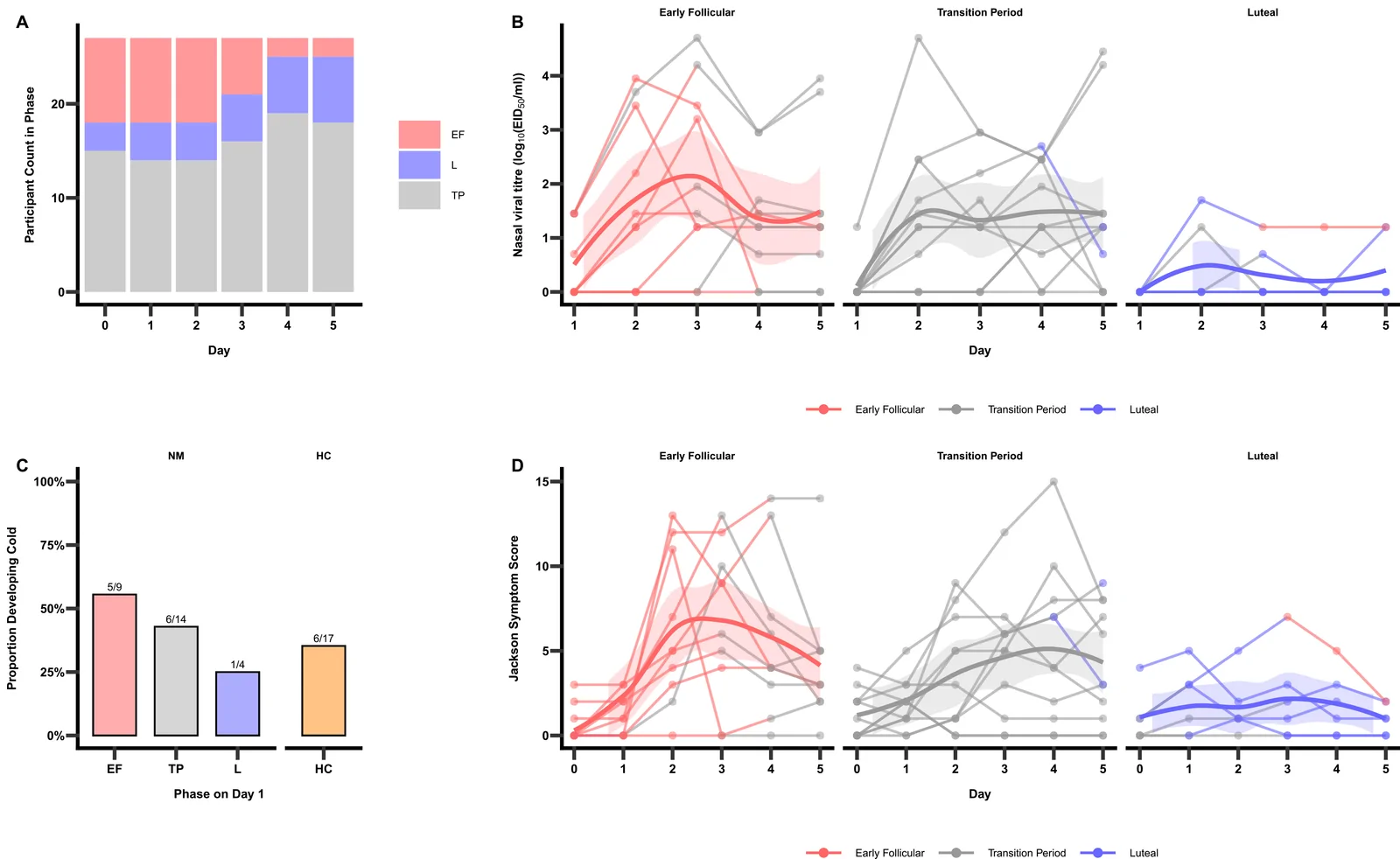
**A**: Proportion and count of phases represented on each day of quarantine within the NM group. EF: estimated early follicular; L: estimated luteal; TP: transition period. **B**: Nasal viral titre during quarantine: participants grouped by dominant phase; individual trajectories show colour-coded transitions between phases. Smoothed line represents a LOESS fit to log_10_-transformed nasal titres (equivalent to the geometric mean trend); shaded bands are 95% confidence intervals. Statistical models used phase as a predictor but these figures illustrate overall trends in nasal titre trajectories clustered by dominant phase. **C:** Objective cold incidence within naturally menstruating participants by menstrual phase on quarantine day 1, versus hormonal contraception (HC) users. Numbers above bars show cases/total participants in each group. **D**: Jackson Common Cold symptom trajectories during quarantine between participants grouped by dominant phase. Smoothed line and bands as in B. (For interpretation of the references to colour in this figure legend, the reader is referred to the Web version of this article.)

### 2.1 Common cold incidence and severity in NM compared to HC

There was no difference between NM and HC for objective cold incidence, viral replication, daily and total subjective symptom score or objective severity (incidence: 44 vs 35%, respectively, OR = 0.68 [0.19–2.35], p = 0.549, Fig. 1C; viral replication and subjective and objective severity: Fig. S2 & Table S2). However, there was a trend towards higher peak nasal titres in the NM group compared to HC (p = 0.054, Table S2).

### 2.2 Common cold incidence and severity across menstrual phases

Within the NM group, estimated menstrual cycle phase on quarantine day 1 was not associated with objective cold development (χ^2^_LRT_ = 0.89, p = 0.64; Fig. 1C).

### 2.3 Menstrual phase effects on viral replication

As expected, there was an increase in viral replication in early quarantine, followed by a decline towards the end of quarantine, which was confirmed by significant effects of day and day^2^ (both p < 0.001; Fig. S3A). The L phase had significantly lower viral titres compared to the EF phase at the quarantine mid-point (β = −0.464 [−0.833 to −0.096], p = 0.042; Fig. 1B, Fig. S3A). Participants who were mostly within the EF phase during quarantine also had higher peak viral titres compared to those predominantly within the L phase (p = 0.003, d = 1.71, Fig. 2A), with Bayesian factor analysis providing moderate evidence that peak nasal titre was higher in EF compared to L (BF_10_ = 8.88). A leave-one-out sensitivity analysis confirmed that the difference in peak viral titre between dominant EF and L phases was robust to exclusion of any single participant (p range: 0.0005–0.0074; mean difference range: 2.01–2.60 log_10_ EID_50_/ml).

**Fig. 2:**
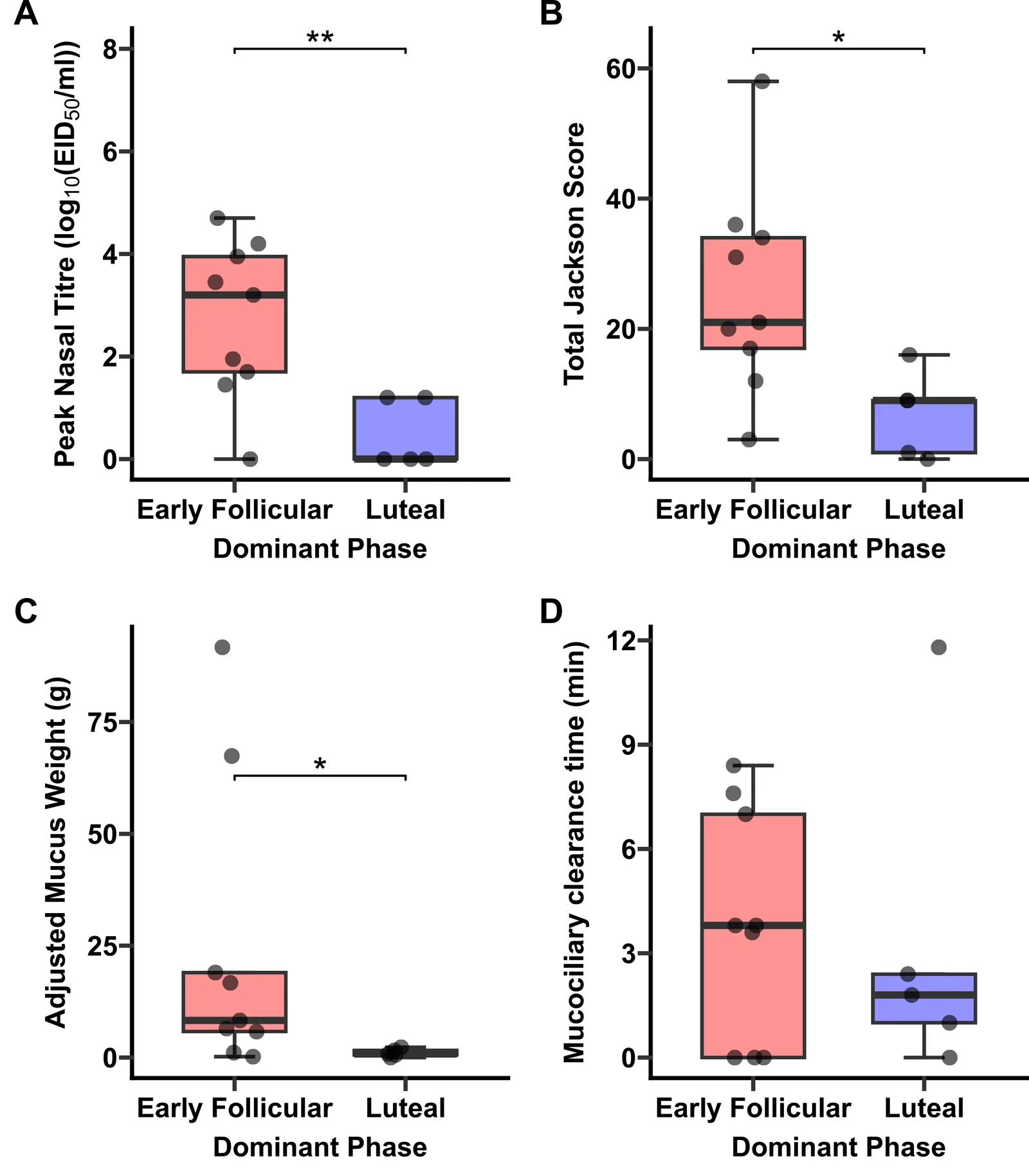
(A) Peak nasal titre during quarantine; (B) Total summed Jackson Common Cold symptom score; (C) objective symptoms assessed by total adjusted nasal mucus secretion rate and (D) nasal mucociliary clearance time between participants grouped by dominant phase. Significant difference between groups, *, p < 0.05, **, p < 0.01.

### 2.4 Menstrual phase effects on symptom progression

In alignment with viral replication, subjective symptoms also followed a biphasic trajectory whereby symptoms increased in early quarantine followed by a decline (effects of day and day^2^, both p < 0.001; Fig. S3B). During the EF phase, symptoms increased over time (β = 0.37 [0.18 – 0.57], p < 0.001), then declined (β = −0.245 [−0.374 to −0.116], p < 0.001) whereas during the L phase, this trajectory was attenuated, resulting in near-stable symptoms during quarantine (β = −0.46 [−0.76 to −0.16], p < 0.01) and an inverted curvature (β = 0.263 [−0.76 to −0.16], p < 0.01; Fig. 1D; Fig. *S3B*). Furthermore, total symptom burden was higher in participants whose estimated dominant phase during quarantine was EF compared to L (p = 0.01, d = 1.37; Fig. 2B). This difference was robust to exclusion of any single participant (p range: 0.0043–0.0248), and equated to moderate evidence according to Bayesian factor analysis (BF_10_ = 3.14). When considering objective cold symptoms, total adjusted mucus weight was higher in EF than L (p = 0.03, d = 0.86, Fig. 2C), although sensitivity analysis indicated this result was less stable, with significance lost upon exclusion of 3 individual participants in the L phase (p range: 0.006–0.076). Nasal mucociliary clearance time did not significantly differ between EF and L (p = 0.74, Fig. 2D).

## 3 Discussion

In this retrospective analysis of data from an experimental viral inoculation study, we found that within NM participants, the luteal phase was associated with attenuated viral replication and symptom development during quarantine compared to the early follicular phase, which represents a novel finding worthy of further exploration. However, we were not able to find evidence of an effect of estimated menstrual cycle phase, or hormonal contraception use, on overall susceptibility to rhinovirus infection, although the small sample size renders these analyses primarily inconclusive. Although preliminary, these observations may have potential implications for women's health and public health. Respiratory viral infections are among the most common illnesses worldwide, and even modest within-person variation in mild respiratory illness severity could influence population health, including health-related quality of life and workplace/school absenteeism (Fragaszy et al., 2018). If menstrual cycle phase contributes to variation in host responses to respiratory viruses, this may represent an under-recognised source of heterogeneity in infection outcomes among premenopausal women and could have implications for future research in immune function and respiratory illness.

The potential for common cold severity to fluctuate throughout the menstrual cycle warrants consideration of potential interactions between the immune system and ovarian hormones, although we acknowledge that ovarian hormone data were not available in the present study. Immune cells express receptors for both oestrogen and progesterone, and meta-analytical data report fluctuations in circulating innate immune cell populations (Notbohm et al., 2023) across the menstrual cycle, but not cytokines or adaptive cell counts. In addition, mucosal immunity in the reproductive tract has been shown to be sensitive to ovarian hormones (Wira et al., 2014). The potential modulation of immune activity across the menstrual cycle may reflect evolutionary trade-offs between maintaining effective immune defence and allowing for reproductive success (Nowak and Muehlenbein, 2025). During the luteal phase, progesterone contributes to creating an immune environment characterised by increased immune tolerance to facilitate implantation and pregnancy (Colamatteo et al., 2026). In the present analysis, we report reduced viral load and symptom severity during the luteal phase, which may be consistent with previous reports of increased immune tolerance (Xu et al., 2023) as well as higher innate immune cells in the luteal phase (Notbohm et al., 2023). Similarly, limited previous work highlights high E2 (as occurs through the mid-cycle and luteal phases) as protective against Covid-19 infection (Ding et al., 2021) and menstruation onset as a potential timepoint of viral reactivation or low resistance to previous infection (Zheng et al., 2021). However, we should not assume that hormones alone may account for the observed patterns, since potential menstrual cycle-related variation in psychosocial, behavioural, and physiological factors may also contribute to respiratory illness severity (Cohen et al., 1991; Ghilotti et al., 2018; Prather et al., 2015).

When considering hormonal contraceptive use, we could not find evidence of HC use influencing overall susceptibility to rhinovirus infection, although the small sample size renders these analyses primarily inconclusive. Similarly, there were no overall differences in viral replication or severity of rhinovirus infection between NM participants and HC users, but we observed a trend towards lower peak viral titres in the HC group. Previous literature supports some role of hormonal contraceptives in modulating systemic immunity (Kjerulff et al., 2026; Tan et al., 2015). However, in contrast to our findings, progesterone-based contraceptives were reported to reduce adaptive immune responses and protect against flu in mice (Hall et al., 2017). These combined observations suggest that further investigation of whether differing hormonal profiles resulting from HC use can influence infection risk and severity is warranted in a larger and better characterised cohort.

The PCS3 dataset provided rich, high-quality experimental data involving rhinovirus inoculation, rarely undertaken due to substantial participant burden, cost and logistical complexity, making these historical data particularly valuable. The experimental design also mitigates concerns about reverse causality given the possible bidirectional relationship between viral infection and the menstrual cycle (Maybin et al., 2025). However, it is important to acknowledge the limitations within this dataset regarding the data on menstrual status and estimated phases. Indeed, eumenorrheic status was not confirmed and hormonal contraceptive type and formulation were not reported. We defined a 10-day luteal phase and five day early follicular phase as conservative phase estimates based on recent population data (Bull et al., 2019), with the remaining days assigned to the transitional period. We also strengthened our approach by excluding participants whose prior menstrual period dates were missing, unclear or unexpected. While this classification by exclusion likely created a more heterogeneous hormonal profile, limiting the ability to attribute biological relevance to these observations, we argue that our approach allows for a more rigorous comparison of outcomes between the early follicular and luteal days. Given that viral burden and symptoms in the early follicular and luteal phases appeared to dichotomise around the mid-cycle (i.e. TP) group, the overall pattern provides some support for the validity of the phase labelling. Nevertheless, the lack of ovarian hormone measurements in the dataset precludes evaluation of cycle irregularities such as anovulatory cycles or luteal phase deficits and may have added noise to our data or led to misclassification of estimated phase. Future research should confirm these findings and any association with oestrogen and progesterone concentrations. The CCP was also seminal in identifying a broad range of psychosocial and behavioural factors that may affect common cold risk (Cohen et al., 1991, 1998, 2013; Prather et al., 2015), but given the already limited statistical power, we did not attempt to account for additional such factors in our analysis. Finally, the age and quality of the dataset may reduce the generalisability of the findings — particularly within the HC group — given the lack of information about HC formulations used by the study participants, and the evolution of contraceptive practices in the USA over time, including increased uptake of hormonal intrauterine devices and implants (Kavanaugh and Jerman, 2018). Consequently, readers should be aware that the hormonal profiles of the HC group may not fully reflect those of contemporary HC users.

Although the original CCP dataset is comprised of data from >750 women, strict inclusion criteria for available variables limited the main screening cohort to the PCS3 dataset, and eventually resulted in very small sample sizes, with only 27 naturally menstruating women included, of whom five were estimated to be predominantly in the luteal phase during quarantine. This renders the analyses presented as vulnerable to low statistical power, with particularly limited ability to assess potential effects of phase on objective cold incidence after experimental rhinovirus exposure, which was our primary *a priori* aim. Given the exploratory nature of our analysis, the findings should therefore be considered primarily hypothesis-generating unless replicated by adequately powered prospective studies. Nevertheless, the reduction in viral load and symptom severity during the luteal phase triangulated well across our linear mixed models, summary contrasts, sensitivity analyses and Bayesian tests, and broadly aligned with patterns observed by others (Davis et al., 2021; Zheng et al., 2021), lending some support to our observation of a significant difference in peak viral load and symptom burden between the luteal and early follicular phases, albeit limited to this small cohort. We hope therefore that these findings offer a springboard to justify further research in this area.

## 4 Conclusion

This novel analysis of historical experimental data indicates that menstrual cycle phase may influence the severity of viral respiratory infections in otherwise healthy, naturally menstruating women. Specifically, we observed a lower viral and symptom burden in the luteal phase and a higher viral and symptom burden during the early follicular phase. These exploratory and hypothesis-generating findings highlight a need to develop improved understanding of how immune function and viral infection susceptibility may fluctuate throughout the menstrual cycle in healthy premenopausal women and thus serve as a call to action for further research to better understand this phenomenon, with a particular need for well-powered studies to assess the influence of menstrual cycle phase and ovarian hormones on susceptibility to common viral illnesses.

## CRediT authorship contribution statement

**Helen G. Hanstock:** Writing – review & editing, Writing – original draft, Visualization, Validation, Software, Formal analysis, Conceptualization. **Sophie E. Harrison:** Writing – review & editing, Validation, Conceptualization.

## Ethics approval

Participants provided written informed consent prior to taking part in the original research study. The study was approved by both the Carnegie Mellon University and University of Pittsburgh Internal Review Boards. All data provided via the Common Cold Project registry were deidentified.

## Availability of data and materials

The data used for analysis were collected by the Laboratory for the Study of Stress, Immunity, and Disease at Carnegie Mellon University under the directorship of Sheldon Cohen, PhD during the years 2005-2011, and were accessed via the Common Cold Project website (www.commoncoldproject.com; https://doi.org/10.17616/R3RS76). All analysis code is available at https://github.com/helenhanstock/ccp_mc.

## Declaration of generative AI and AI-assisted technologies in the manuscript preparation process

During the preparation of this work the author (HH) used large language models (LLMs) to develop and troubleshoot R syntax, including Claude (Anthropic) and Copilot (Microsoft). After using these services, the authors reviewed and edited the code, verified outputs and take full responsibility for the content of the published article.

## Funding

CCP data were made available via a grant from the National Center for Complementary and Integrative Health (NCCIH AT006694). The PCS3 study data were acquired thanks to funding from the National Institute of Allergy and Infectious Diseases (NIAID R01 AI066367). Secondary funding was obtained from the National Institutes of Health (NIH UL1 RR0024153 and UL1 TR0005). Supplementary measures were funded by the Pennsylvania Department of Health through a Commonwealth Enhancement Grant (08-01-2), the National Centre for Complementary and Integrative health (RC1AT005799), the John D and Catherine T MacArthur Foundation Research Network on Socioeconomic Status & Health.

The authorship team have no additional funding sources to declare.

## Declaration of competing interest

The authors declare that they have no known competing financial interests or personal relationships that could have appeared to influence the work reported in this paper.
