# Supplementary material for: Effect of the menstrual cycle on common cold susceptibility and severity: insights from the Pittsburgh cold study 3 dataset
Source: Brain Behav Immun Health. 2026 Nov 4;58:101402. doi: 10.1016/j.bbih.2026.101402 (PMC13763571; doi:10.1016/j.bbih.2026.101402)
Supplement: Supplementary file 1 [file mmc1.docx]

**Supplementary Materials**

**Supplementary Table 1:** Participant and group characteristics

| **Characteristic** | **Overall (N=44)** | **NM (N=27)** | **HC (N=17)** |
| --- | --- | --- | --- |
| Age (years) | 24 (5) | 24 (5) | 24 (6) |
| BMI (kg/m^2^) | 26 (7) | 27 (7) | 25 (6) |
| Self-reported bleed duration (days) | 5 (1) | 5 (1) | 5 (1) |
| Self-reported cycle length (days) | 28 (2) | 28 (2) | 28 (2) |
| Self-reported health rating |  |  |  |
| - Fair | 7 (16%) | 5 (19%) | 2 (12%) |
| - Good | 25 (57%) | 15 (56%) | 10 (59%) |
| - Excellent | 12(27%) | 7 (26%) | 5 (29%) |
| Actigraphy-assessed sleep |  |  |  |
| - < 5 h | 5 (14%) | 3 (14%) | 2 (14%) |
| - 5 – 6 h | 14 (39%) | 8 (36%) | 6 (43%) |
| - 6 – 7 h | 11 (31%) | 6 (27%) | 5 (36%) |
| - > 7 h | 6 (17%) | 5 (23%) | 1 (7%) |
| - missing | 8 | 5 | 3 |
| Self-reported days/week with physical activity | 4 (3) | 4 (2) | 4 (3) |

*Values are mean (SD) or n (%)*

**Supplementary Table 2:** Viral titres, symptoms and objective cold signs during quarantine in the naturally menstruating (NM) and hormonal contraceptive (HC) groups.

| **Variable** | **Overall (N=48)** | **NM (N=27)** | **HC (N=17)** | **p-value***^2^* |
| --- | --- | --- | --- | --- |
| Peak nasal titre (log₁₀(EID₅₀/ml)) | 1.45 (0.50, 2.58) | 1.70 (1.20, 3.20) | 1.00 (0.00, 2.00) | 0.054 |
| Total Jackson Symptom Score | 17 (11, 29) | 17 (9, 31) | 15 (12, 27) | 0.990 |
| Total adjusted Post-challenge mucus weight (g) | 6 (1, 18) | 6 (1, 17) | 6 (0, 22) | 0.735 |
| Avg Adjusted Post Nasal Clearance Time (min) | 1.8 (0.2, 6.6) | 2.0 (1.0, 6.6) | 1.6 (0.0, 6.6) | 0.817 |

*Values are median and quartiles (1, 3). P value represents results from Wilcoxon signed-rank test comparing HC and NM.*

***Supplementary Figure 1****. Flow diagram of study inclusion and data analysis for hormonal contraceptive (HC) and naturally menstruating (NM) participants. EF, early follicular phase. L, luteal phase. TP, transitional period.*

***Supplementary Figure 2.*** *Nasal titres (A), and symptom scores (B) in hormonal contraception (HC; orange) users versus naturally menstruating (NM) participants (pink). Data are A) geometric mean and 95% confidence interval bands and B) mean and 95% confidence interval bands.*

***Supplementary Figure 3.*** *Model fit for linear mixed models investigating effect of quarantine day and phase on a) viral nasal titres and b) symptom scores. In both cases, y axis variables are log10(variable+1) transformed. To model viral load trajectories during the five post-inoculation quarantine days, we compared two models: a linear model (AIC = 195; BIC = 218; R^2^m = 0.119) and a quadratic model including a phase × day² interactions (AIC = 195; BIC = 227, R^2^m = 0.177), with the quadratic model selected. To model symptom trajectories, we compared again a linear model (AIC = 300, BIC = 327, R^2^m = 0.215) and a quadratic model (AIC = 298, BIC = 333, R^2^m = 0.272), with the quadratic model selected. EF, early follicular phase. L, luteal phase. TP, transitional period.*
